# Work as a Predictor of Ethylenethiourea (ETU) Exposure During Pregnancy Among Participants Enrolled in the SEMILLA Birth Cohort Study

**DOI:** 10.3390/toxics13110988

**Published:** 2025-11-17

**Authors:** Alexis J. Handal, Fadya Orozco, Stephanie Montenegro, Nataly Cadena, Fabián Muñoz, Eileen Ramírez del Rio, Niko Kaciroti

**Affiliations:** 1 Department of Epidemiology, School of Public Health, University of Michigan, Ann Arbor, MI 48109-2029, USA; eilram@umich.edu; 2 Centro de Transferencias y Desarrollo de Tecnologías CTT-USFQ, Universidad San Francisco de Quito USFQ, Quito 170157, Ecuador; fady5o@yahoo.es (F.O.); stefymv@gmail.com (S.M.); natalycadenam92@outlook.com (N.C.); 3 Visor Análisis Estadístico Cia. Ltd., Quito 170150, Ecuador; fabian_munoz@yahoo.com; 4 Department of Pediatrics, School of Medicine, University of Michigan, Ann Arbor, MI 48109-2029, USA; nicola@umich.edu; 5 Department of Biostatistics, School of Public Health, University of Michigan, Ann Arbor, MI 48109-2029, USA

**Keywords:** endocrine disruptors, epidemiology, maternal and fetal exposure/health, pesticides, workplace exposures, Latin America, birth cohort, community-engaged research

## Abstract

Background: Ethylenebisdithiocarbamates, widely used in floriculture, degrade into ethylenethiourea (ETU), a teratogen. The SEMILLA study investigates prenatal ETU exposure and infant health in Ecuador’s flower-growing region. This analysis examines whether prenatal ETU metabolite levels differ by work sector and whether maternal urinary ETU increases with longer work hours. Methods: Participants (agricultural workers, non-agricultural workers, and non-workers) provided baseline urine samples, which were processed and stored for ETU analysis. Surveys captured ETU exposure predictors. Regression models assessed associations between work sector, weekly work hours, and urinary ETU levels (specific gravity-corrected), controlling for key covariates. Results: The sample includes 111 agricultural workers (92% floriculture), 149 non-agricultural workers, and 149 non-workers. At baseline, maternal age averaged 27 years (SD = 5.8) and gestational age 15 weeks (SD = 3.2). Urinary ETU_SG_ levels were elevated across the sample (geometric mean: 3.38 µg/L). Agricultural workers had significantly higher ETU_SG_ levels than others (5.61 vs. 3.07 and 2.57 µg/L; *p* < 0.001). Among agricultural workers, ETU_SG_ levels increased with weekly hours (B = 0.288, *p* = 0.001). Conclusions: Agricultural work strongly predicts higher prenatal ETU exposure, with evidence of a dose–response relationship. Research on prenatal fungicide exposure and infant health among pregnant workers is limited. Findings highlight the need for targeted interventions to protect pregnant workers and infant health.

## 1. Introduction

Industrial agriculture plays an important role in the global economy and accounts for a substantial percentage of global pesticide use [1]. Fungicides are now the most commonly used pesticides worldwide. Their use has exploded over the past decade, with a predicted US$32.3 billion by 2029 [2,3]. In the United States (U.S.) and other high-income countries, where the majority of research is often conducted, fungicidal metabolites are not widely studied in humans because exposure levels in these countries have historically been non-detectable [4]. However, with the boom in use of fungicides worldwide, particularly in low- and middle-income countries (LMICs), research on the impact of these fungicides on human health has become a priority. Here, we present findings on prenatal fungicide exposure from participants enrolled in the birth cohort study, SEMILLA (Study of Environmental Exposure of Mothers and Infants Impacted by Large-Scale Agriculture).

### Background and Rationale

Ethylenebisdithiocarbamates, or EBDCs, are commonly used fungicides in agricultural production as well as in floriculture and horticulture [5]. EBDCs are classified by the World Health Organization as “U” (unlikely to pose an acute hazard in normal use) [6]. However, the U.S. Environmental Protection Agency considers EBDCs, along with their more toxic degradation product, ethylenethiourea (ETU), as probable human carcinogens [7,8]. Furthermore, ETU is considered a toxic teratogenic compound. In animal models, ETU has been associated with central nervous system teratogenicity (such as brain malformations) [9,10], and neurological impairments such as hyperactivity, ataxia, and hindlimb paralysis [11]. Further, animal studies show that ETU is an anti-thyroid compound and can impact fetal development and infant thyroid function [8,12], even at low doses [13].

Few human studies have focused specifically on exposure to EBDC fungicides, and specifically to ETU. A recent systematic review of human biomonitoring studies highlights the limited and outdated data on ETU exposure in various populations, with most studies being small, over a decade old, and focused on populations with known EBDC exposure such as adult agricultural workers, with minimal data focused on pregnant workers and those in LMICs [4]. Studies assessing the health impacts of ETU exposure have mainly focused on male agricultural workers [14,15,16,17,18]. Montesano and Wang at the CDC provide a comprehensive review of these findings [5]. A small number have focused on women in agricultural settings. The Agricultural Health Study reported an association between hypothyroidism and mancozeb exposure among spouses of farmworkers [19]. In Florida, ETU levels among pregnant and non-pregnant fernery and nursery workers were comparable to other occupational cohorts and higher than the general population [geometric mean: 1.90 μg/L and 5.69 μg/L, respectively] [20]. In Costa Rica, high urinary levels of ETU were documented in pregnant women residing near large-scale banana plantations, where use of EBDC fungicides is widespread [Geometric mean (1st trimester): 3.1 μg/L, (IQR) = 1.8–4.6] [21].

The Ecuadorian flower industry is representative of the growing agricultural export industry and provides an opportunity to evaluate occupational and environmental exposure to fungicides in agricultural communities and workers. EBDCs are commonly used fungicides in agricultural production in Ecuador [22]. The Ecuadorian flower industry is no exception. In Ecuador, flowers have become the country’s sixth most important export. In 2021, roughly US$937 million worth of cut flowers were exported (up ~7% from 2019), with ~43% exported to the United States [23]. Pesticide use in this industry is widespread, including the use of fungicides such as the EBDCs. Handal and colleagues previously documented high levels of prenatal ETU metabolite levels [geometric mean: 3.73 μg/L] among participants in a pilot study assessing the feasibility of quantifying prenatal pesticide levels in pregnant Ecuadorian women residing near or working in industrial floriculture greenhouses [24].

The SEMILLA birth cohort study investigates possible adverse effects of prenatal ETU exposure on newborn thyroid function and infant growth and neurobehavioral development in a major flower-growing region of Ecuador. This paper identifies potential predictors of elevated prenatal maternal urinary ETU levels at baseline, with maternal work during pregnancy as the main predictor.

## 2. Materials and Methods

### 2.1. Study Design

The SEMILLA study is a prospective longitudinal birth cohort study conducted in a major agricultural region of Ecuador. Led by researchers from the University of Michigan and the Universidad San Francisco de Quito, this study follows pregnant women and their infants over approximately two years to observe the effects of early exposures on child development [25].

### 2.2. Study Setting

The study is based in the Pichincha province, specifically in the cantons of Cayambe and Pedro Moncayo, located about 90 km northeast of Quito, in the northern highlands region of the country. This region, with an estimated population of 118,967, has seen a significant increase in greenhouse cut-flower production, employing a large female workforce of reproductive age. The region’s demographics and economic activities make it an ideal location for studying occupational and environmental exposures.

This region was selected for several reasons: (1) it has seen a dramatic decade-long increase in industrial greenhouses used for flower production and concentrates about half of the country’s flower production [26,27]; (2) the flower industry employs a large female labor force, mainly of reproductive age; (3) residential communities are in close proximity to the industrial greenhouses; and (4) it is where the investigators have successfully conducted previous studies that informed the aims of SEMILLA [24,28,29,30].

### 2.3. Study Population

Participants include pregnant women from the Cayambe and Pedro Moncayo cantons. Eligibility criteria were adapted from prior research conducted in similar populations and included the following: age 18 years or older, gestational age between 8 and 20 weeks, continuous residence in the study area for at least one year prior to enrollment, and an intention to remain in the area for at least one year following delivery. Given previous evidence of elevated ETU metabolite levels among both agricultural and non-agricultural workers [24], participants were categorized into three employment-based groups: agricultural workers, non-agricultural workers, and non-workers. The inclusion of agricultural workers allowed for the evaluation of potential occupational ETU exposure within the agricultural sector. Incorporating non-agricultural workers and non-workers provided a broader understanding of occupational and environmental exposures at the community level. This design also facilitated examination of key contextual factors, such as psychosocial stress and social support, which may differ by employment status and influence exposure or health outcomes.

### 2.4. Recruitment, Enrollment, and Follow-Up

The study aimed to recruit 420 participants, stratified by maternal occupational status. Due to COVID-19 and political unrest, the recruitment period spanned 30 months (October 2019 to April 2022). Diverse outreach strategies included collaboration with health professionals, obstetric census, non-monetary incentives through institutional and community spokespeople. Women expressing interest in the study first completed eligibility screening, which included a confirmatory ultrasound to verify a viable pregnancy between 8 and 20 weeks of gestation. Those meeting the inclusion criteria were then invited to complete the informed consent process, led by the Technical Coordinator, who reviewed consent forms and addressed any questions before obtaining written consent from participants for themselves and their infants.

The field team consisted of the Technical Coordinator, recruiters, and trained Community Workers responsible for participant recruitment, tracking, and interviews. Regular phone calls and home visits helped maintain contact and build trust with participants. Additional strategies, such as incentives and professional advice in key areas such as maternal health, nutrition, child stimulation, were implemented to reduce losses to follow-up, ensuring high retention rates throughout the study. This comprehensive approach, from recruitment to follow-up, ensured the study effectively captured the impact of early exposures on child development in a unique agricultural setting.

### 2.5. Urinary Ethylenethiourea (ETU) Data Collection and Measurement

Ethylenethiourea (ETU) metabolite concentration levels during pregnancy were assessed using prenatal first morning void (FMV) urine samples collected up to 3 times over the pregnancy. Participants were enrolled between 8 and 20 weeks of gestation and provided up to three FMV spot urine samples during pregnancy, with the number of samples varying according to gestational age at the time of enrollment. After providing a short training session to participants on how to collect a FMV urine sample, participants were provided with a 120 mL sterile container to collect the sample. Samples were collected at home within 1 week of the survey interview. Samples were then transferred in a cooler by the field team for processing into 15 mL cryovials containing 3 mL of urine and stored at −20 °C at the study office in Cayambe, until shipment to laboratory partners at the National Institute of Public Health of Quebec (the Centre de Toxicologie du Québec [31]) for analysis of urinary ETU metabolite concentration levels.

### 2.6. Chemical Analyses of ETU in Urine

Urinary analyses of ethylenethiourea (ETU) were performed by the Centre de Toxicologie du Québec (CTQ) of the Institut national de santé publique du Québec (INSPQ). The CTQ has established protocols for the measurement of ETU. Briefly, the analytical method was performed as follows: 100 μL of urine samples were enriched with labeled internal standard (ETU-d4) and hydrolyzed with 30 µL of a 10 M NaOH solution. Thereafter, the samples were derivatized with pentafluorobenzyl bromide (PFBBr) for 90 min in an ultrasonic bath before being extracted by a liquid–liquid technique with 2 mL of hexane. The extracts were evaporated to dryness, reconstituted in 1 mL of acetonitrile and a fraction (20 µL) of the latter was dissolved in 1 mL of 1 mM ammonium acetate in 60% acetonitrile. The extracts were analyzed by Ultra Performance Liquid Chromatography (UPLC Waters Acquity) with a tandem mass spectrometer (MS/MS Waters Xevo TQ-S) (Waters, Milford, MA, USA) in MRM mode with an electrospray ion source in the positive mode. The column used was an ACE EXCEL C18 50 × 2.1 mm, 2.0 µm (ACE; Aberdeen, Scotland). The mobile phase consisted of a gradient of 5 mM ammonium acetate/acetonitrile (40:60) to 100% of acetonitrile in 3.0 min with a flow rate of 0.5 mL/minute. The LOD was 0.033 μg/L for ETU. The intra-day precision was 6.9% and the inter-day precision was 7.9%. A calibration curve up to a concentration of 100 μg/L was prepared in urine for quantification. The internal reference materials used to control the quality of the analyses were 4 levels (Near LOQ, Low, Med, High) of in-house quality controls (QCs) prepared from an ETU standard source different from the calibration curve. There is no quality assessment scheme available for this analyte. Urine data were not creatinine-adjusted because of the physiologic changes in pregnancy that increase the intraperson variation in creatinine excretion, thus rendering it unsuitable for normalizing urine dilution [32]. However, urine data were quantified for dilution in two other ways: (1) by design by collecting FMV samples; (2) measuring urine samples for specific gravity. Specific gravity (SG) was measured upon urine sample collection using a handheld digital refractometer (ATAGO, PAS-10S, Bellevue, WA, USA).

### 2.7. Predictors of ETU

The present analysis focused on predictors of ETU levels in this population of pregnant workers and non-workers. Namely, the main predictor of ETU comprised work-related ETU exposure, including work sector at the time of the baseline interview (i.e., whether the participant worked in agriculture, in non-agricultural work, or was not working outside of the home for pay at the time of the baseline interview). In addition, we considered other key potential covariates, collected by survey administered to the participant at the baseline visit. Data were collected on: sociodemographic and maternal characteristics such as maternal age, gestational age, partner’s work sector, number of people living in the home, monthly income, home and material ownership; residential characteristics such presence of domestic plots/vegetable patches, presence of nearby irrigation ditches or open water canals and distance to nearby ditches/canals, air quality, and frequency of consumption of foods with high potential of fungicide use (potato, kidney and tree tomato) over the past week. Data on key occupational characteristics were also collected via survey, including contact with chemical products at work during the past week, access to water at work for hand hygiene, access to soap at work, access to alcohol/gel at work for hand hygiene.

### 2.8. Statistical Analysis

This analysis focuses on the baseline (enrollment) time point to test the following two hypotheses: (1) whether the exposure to type of work sector affects urinary ETU metabolite levels (agriculture workers vs. non-agriculture workers vs. non-workers); and (2) whether among workers (agriculture and non-agriculture), there is a dose response relationship with longer work hours/week being associated with higher levels of urinary ETU and whether such relationship varies by the work sector.

To improve the normality assumption and reduce skewness on the distribution of ETU, we utilized a logarithm-transformed ETU to test for log-linear trends using general linear models. In addition, we conducted multiple logistic regression analyses using a binary outcome variable for ETU exposure, defined as high ETU levels (>4 μg/L), to assess the potential presence of a threshold effect. The >4 μg/L cut-off was selected based on two considerations: (1) approximately 40% of the sample exceeded this level, providing a more balanced distribution for statistical analysis; and (2) previous studies conducted in the region, including our own [24], as well as research in similar populations [20,21], have reported geometric mean ETU levels around 3 μg/L among pregnant and non-pregnant female workers. We also tested a 3 μg/L threshold, and results were similar to those using 4 μg/L. We ultimately selected 4 μg/L to maintain more even exposure group sizes and to explore a threshold that may reflect relatively higher exposure. Importantly, there is currently no established health-based threshold for ETU, limiting the clinical interpretability of any specific cut-off.

We examined both specific gravity corrected and uncorrected urinary ETU concentration levels for statistical modeling purposes. Given that findings were similar, we utilized specific gravity corrected ETU concentration levels in the analysis, based on the correction methods outlined by MacPherson and colleagues [33]. Corrected analyte concentrations were obtained using the following formula:

CHEM_SG_adj_ = CHEM*_i_* ((SG*_m_*− 1)/(SG*_i_* − 1))

Here, CHEM_SG_adj_ is the specific gravity adjusted analyte concentration, CHEM*_i_* is the original (unadjusted) concentration, SG*_m_* is the median specific gravity for the specific cohort being observed, and SG*_i_* is the specific gravity of the urine associated with the analyte concentration. The corrected ETU measure is reported hereafter as ETU_SG_.

First, we ran unadjusted analyses of sociodemographic and economic characteristics, residential environment, and maternal labor history characteristics for the total sample and stratified by work sector, using Fisher’s exact test for categorical variables and Wilcoxon’s rank sum test for continuous variables. Multivariate regression models were implemented for adjusted analysis controlling for potential confounders. Confounders considered for inclusion were selected based on a combination of theoretical and empirical considerations. Specifically, we relied on conceptual models informed by our previous work in the region and supported by the existing literature, as well as initial statistical testing. The full model included variables identified a priori as relevant to ETU exposure, encompassing both residential and occupational factors (see Table 1). Our primary predictor of interest was work sector at baseline (i.e., agricultural work, non-agricultural work, or not working outside the home). After specifying the full model, we used a manual stepwise selection procedure, with entry and retention criteria set at *p* < 0.05, to refine the model and identify the most influential covariates. We obtained the most parsimonious models by removing those covariates that were not significantly related to the outcome (ETU). While we acknowledge the limitations of stepwise selection, including the potential for overfitting and biased estimates, it was used here to complement, not replace, our theory-driven approach and to explore the relative contribution of the covariates in this dataset.

Statistical significance was set using a two-sided *p*-value < 0.05. Two set of models were run, one using urinary ETU concentration as a continuous measure log-transformed to reduce the skewness and improve normality for the model residuals, and a binary categorical urinary ETU concentration measure was defined as ≤4 μg/L (lower exposure) and >4 μg/L (higher exposure), based on the distribution in the sample and previous literature identifying this cut-off as a potentially important value for health effects, as previously noted.

We used a general linear model (GLM) to test whether urinary ETU_SG_ levels differ by work sector (hypothesis 1), adjusting for potential confounders. Non-significant predictors were removed resulting in a parsimonious model. The final model included the participants’ work sector as the main predictor and other covariates that were significantly related to the outcome. In addition, we fitted a multivariate logistic regression to test whether work sector was related to high levels of ETU_SG_ (defined as ETU_SG_ > 4 μg/L). The same variables from the previous model were initially included, with the final model containing the participants’ work sector and other significant covariates.

For the second hypothesis, we used GLM on the workers only sample to test whether there was a dose–response relationship between work hours and ETU_SG_ levels including work hours as an additional predictor in the model. In addition, we included an interaction term of work hours and work sector to test whether work sector modified the dose–response relationship between work hours and ETU_SG_ levels. The final model included the participants’ work sector, work hours and their interaction as the main predictors and other covariates that were significantly related to the outcome in this context. Similarly, we fitted a multivariate logistic regression with ETU_SG_ levels greater than 4 μg/L as a binary outcome. Standardized betas are reported for the worker-only model on continuous predictor (work hours) to display the magnitude of association between work hours and log-ETU.

All analyses of urine metabolite levels were run using ETU_SG_ levels corrected for specific gravity, following established protocols and methods [34]. All analyses were run using SAS 9.4 (SAS Institute Inc. 2023. SAS/STAT^®^ 15.3 User’s Guide. Cary, NC, USA: SAS Institute Inc.).

## 3. Results

The SEMILLA study enrolled 409 participants (72% enrollment rate from those eligible to participate). The sample consisted of 111 agricultural workers (92% in floriculture), 149 non-agricultural workers, and 149 non-workers (women not engaged in paid employment outside the home at the time of the baseline interview). Baseline data on urinary ETU metabolite levels were collected on 406 participants (99%); urine samples were not collected on three participants due to the COVID-19 pandemic shutdown at the time of collection.

### 3.1. Sample Characteristics

Table 1 presents the summary of socioeconomic and demographic characteristics (Table 1A), residential environmental (Table 1B), and occupational characteristics (Table 1C) of the SEMILLA cohort study sample at baseline, overall and by maternal work sector. More completed and detailed sociodemographic and economic data of the sample is described elsewhere [25].

**Table 1:** Socioeconomic, demographic, and general environmental and occupational characteristics of the SEMILLA cohort study sample at baseline, by maternal work sector. Cayambe, Ecuador. 2018–2024.

Characteristic	All Participants (*N* = 409) ^1^	Agricultural Workers ^2^ (*N* = 111)	Non-Agricultural Workers (*N* = 149)	Non-Workers (*N* = 149)	*p*-Value
**A. Socioeconomic and Demographic Characteristics**					
**Maternal age**					
M (SD)	27.2 (5.8)	28.7 (5.3)	27.5 (6.2)	25.8 (5.6)	<0.001
**Gestational age**					
M (SD)	15.3 (3.2)	15.1 (3.0)	14.9 (3.3)	15.8 (3.1)	0.053
**Current city of residence *N* (%)**					0.095
Cayambe canton	162 (42.9)	46 (45.1)	61 (43.3)	55 (40.7)	
Cayambe canton (Cayambe urban center)	120 (31.7)	21 (20.6)	51 (36.2)	48 (35.6)	
Pedro Moncayo canton	42 (11.1)	15 (14.7)	13 (9.2)	14 (10.4)	
Pedro Moncayo canton (Tabacundo urban center)	54 (14.3)	20 (19.6)	16 (11.3)	18 (13.3)	
**Length of time living in current city (years)**					
M (SD)	11.5 (11.6)	13.7 (12.4)	11.9 (11.7)	9.3 (10.6)	0.010
**Status of current home *N* (%)**					<0.001
Owned by participant/her husband or partner	107 (26.2)	44 (39.6)	38 (25.5)	25 (16.9)	
Rented/loaned/through services	217 (53.0)	43 (38.8)	75 (50.3)	99 (66.3)	
Owned by parents	85 (20.8)	24 (21.6)	36 (24.2)	25 (16.8)	
**Length of time living in current house (years)**					
M (SD)	6.8 (8.8)	8.7 (10.2)	7.2 (9.1)	5.0 (6.8)	0.002
**Electricity in the home *N* (%)**					0.115
No	3 (0.7)	0 (0.0)	0 (0.0)	3 (2.0)	
Yes	406 (99.3)	111 (100.0)	149 (100.0)	146 (98.0)	
**Material ownership scale**					
M (SD)	7.2 (1.9)	7.4 (1.5)	7.6 (1.9)	6.7 (2.0)	<0.001
**Number of people that live at home**					
M (SD)	4.0 (2.1)	3.8 (1.5)	4.0 (2.2)	4.2 (2.3)	0.377
**Number of people <18 that live at home**					
M (SD)	1.4 (1.2)	1.4 (0.9)	1.3 (1.4)	1.4 (1.3)	0.861
**Lives at home with partner/husband *N* (%)**					0.118
No	95 (23.2)	18 (16.2)	39 (26.2)	38 (25.5)	
Yes	314 (76.8)	93 (83.8)	110 (73.8)	111 (74.5)	
**Lives at home with children *N* (%)**					0.001
No	145 (35.4)	23 (20.7)	62 (41.6)	60 (40.3)	
Yes	264 (64.6)	88 (79.3)	87 (58.4)	89 (59.7)	
**Marital status *N* (%)**					0.0004
Married	107 (26.2)	27 (24.3)	49 (32.9)	31 (20.8)	
Free union	209 (51.1)	65 (58.6)	59 (39.6)	85 (57.0)	
Separated/divorced/widowed	13 (3.1)	7 (6.3)	5 (3.3)	1 (0.7)	
Single	80 (19.6)	12 (10.8)	36 (24.2)	32 (21.5)	
**Language spoken at home *N* (%)**					0.012
Spanish	396 (96.8)	103 (92.8)	147 (98.6)	146 (98.0)	
Quechua	1 (0.3)	0 (0.0)	1 (0.7)	0 (0.0)	
Mix of Spanish and Quechua	12 (2.9)	8 (7.2)	1 (0.7)	3 (2.0)	
**Mother’s educational level (years)**					
M (SD)	12.2 (3.7)	11.0 (3.3)	13.3 (3.9)	12.0 (3.4)	<0.001
**Spouse/partner educational level (years)**					
N	376	105	138	133	
M (SD)	11.7 (3.5)	11.0 (3.1)	12.5 (3.6)	11.4 (3.5)	0.002
**Currently, number of people that work for income at home**					
N	396	107	140	149	
M (SD)	1.9 (1.0)	2.1 (0.9)	2.2 (1.0)	1.3 (0.8)	<0.001
**Total monthly income at home (US $)**					
N	364	99	125	140	
M (SD)	$512 (227)	$636 (201)	$533 (218)	$404 (200)	<0.001
**Number of people that live from monthly income at home**					
M (SD)	4.2 (3.3)	4.5 (4.6)	4.2 (2.9)	4.0 (2.4)	0.487
**Primary person responsible for generating a majority of income at home *N* (%)**					<0.001
Participant	65 (15.9)	22 (19.8)	42 (28.2)	1 (0.7)	
Husband/partner	232 (56.7)	54 (48.7)	72 (48.3)	106 (71.1)	
Both earn the same	36 (8.8)	24 (21.6)	11 (7.4)	1 (0.7)	
Parents	42 (10.3)	4 (3.6)	18 (12.1)	20 (13.4)	
Other	22 (5.4)	7 (6.3)	5 (3.3)	10 (6.7)	
Not applicable (no one)	12 (2.9)	0 (0.0)	1 (0.7)	11 (7.4)	
**Financial hardship scale**					
M (SD)	1.8 (1.8)	1.5 (1.6)	1.6 (1.8)	2.2 (1.7)	0.005
**Mother’s self-reported ethnicity *N* (%)**					0.241
Indigenous	88 (21.5)	31 (27.9)	29 (19.5)	28 (18.8)	
Mestiza	314 (76.8)	78 (70.3)	116 (77.8)	120 (80.5)	
Afro-Ecuadorian	3 (0.7)	1 (0.9)	1 (0.7)	1 (0.7)	
White	4 (1.0)	1 (0.9)	3 (2.0)	0 (0.0)	
**Partner’s ethnicity (reported by participant) *N* (%)**					0.403
Indigenous	69 (16.9)	25 (22.5)	23 (15.4)	21 (14.1)	
Mestizo	298 (72.9)	80 (72.1)	109 (73.2)	109 (73.2)	
Afro-Ecuadorian	1 (0.2)	0 (0.0)	1 (0.7)	0 (0.0)	
White	6 (1.5)	1 (0.9)	3 (2.0)	2 (1.3)	
Does not know/does not remember	7 (1.7)	2 (1.8)	2 (1.3)	3 (2.0)	
Not applicable (no partner)	28 (6.9)	3 (2.7)	11 (7.4)	14 (9.4)	
**B. Residential Environmental Characteristics**					
**Type of hygienic service at home (toilet) *N* (%)**					0.034
None	3 (0.7)	1 (0.9)	1 (0.7)	1 (0.7)	
Toilet with septic tank	80 (19.6)	31 (27.9)	20 (13.4)	29 (19.4)	
Toilet with sewage system	325 (79.5)	79 (71.2)	128 (85.9)	118 (79.2)	
Doesn’t know/does not remember	1 (0.2)	0 (0.0)	0 (0.0)	1 (0.7)	
**Type of water consumed at home *N* (%)**					0.777
Open well	2 (0.5)	1 (0.9)	1 (0.7)	0 (0.0)	
Piped, non-potable (inside/outside of home)	12 (2.9)	2 (1.8)	4 (2.6)	6 (4.0)	
Piped potable water	393 (96.1)	108 (97.3)	143 (96.0)	142 (95.3)	
Tank delivery by truck/bottled	2 (0.5)	0 (0.0)	1 (0.7)	1 (0.7)	
**Type of water for domestic use *N* (%)**					0.685
Open well	2 (0.5)	1 (0.9)	1 (0.7)	0 (0.0)	
Piped, non-potable (inside/outside of home)	14 (3.4)	2 (1.8)	6 (4.0)	6 (4.0)	
Piped potable water	392 (95.9)	108 (97.3)	142 (95.3)	142 (95.3)	
Tank delivery by truck/bottled	1 (0.2)	0 (0.0)	0 (0.0)	1 (0.7)	
**Irrigation ditch/open water canal nearby *N* (%)**					0.977
No	312 (76.3)	85 (76.6)	112 (75.2)	115 (77.2)	
Yes	95 (23.2)	26 (23.4)	36 (24.1)	33 (22.1)	
Doesn’t know/does not remember	2 (0.5)	0 (0.0)	1 (0.7)	1 (0.7)	
**Distance to irrigation ditch/water canal (meters)**					0.058
N	75	21	28	26	
M (SD)	16.7 (17.3)	24.2 (21.0)	14.7 (14.2)	12.9 (15.7)	
**Has plots/vegetable patch *N* (%)**					0.636
No	268 (65.5)	70 (63.1)	96 (64.4)	102 (68.5)	
Yes	141 (34.5)	41 (36.9)	53 (35.6)	47 (31.5)	
**Primary person responsible for the plots/vegetable patch *N* (%)**					0.710
Participant	50 (35.5)	14 (34.1)	18 (34.0)	18 (38.3)	
Participant’s husband/partner	34 (24.1)	12 (29.3)	14 (26.4)	8 (17.0)	
Other	57 (40.4)	15 (36.6)	21 (39.6)	21 (44.7)	
**Use of chemical products for the plots/vegetable patch *N* (%)**					0.026
No, never	121 (85.8)	30 (73.2)	49 (92.5)	42 (89.4)	
Yes	20 (14.2)	11 (26.8)	4 (7.5)	5 (10.6)	
**During the past week, frequency of potato consumption**					
M (SD)	7.1 (5.8)	8.1 (6.4)	7.2 (5.7)	6.4 (5.5)	0.075
**During the past week, frequency of kidney tomato consumption**					
M (SD)	4.8 (4.1)	4.9 (4.3)	5.1 (4.1)	4.5 (3.8)	0.483
**During the past week, frequency of tree tomato consumption**					
M (SD)	1.7 (2.8)	1.6 (2.4)	1.8 (2.9)	1.8 (2.9)	0.702
**Air quality outside of the home *N* (%)**					0.958
Clean, without odor	214 (52.3)	57 (51.4)	79 (53.0)	78 (52.4)	
Other	195 (47.7)	54 (48.6)	70 (47.0)	71 (47.6)	
**C. Occupational Characteristics**					
**During the past week, number of weekly work hours**					0.491
N	260	111	149	-	
M (SD)	38.5 (18.8)	39.4 (16.4)	37.8 (20.3)	-	
**During the past week, contact with chemical products at work *N* (%)**					<0.001
No	218 (83.9)	81 (73.0)	137 (92.0)	-	
Yes	42 (16.1)	30 (27.0)	12 (8.0)	-	
**Access to water at work for hand hygiene *N* (%)**					0.282
Most of the time	242 (93.0)	102 (91.9)	140 (94.0)	-	
Sometimes	9 (3.5)	3 (2.7)	6 (4.0)	-	
Never	9 (3.5)	6 (5.4)	3 (2.0)	-	
**Access to soap at work for hand hygiene *N* (%)**					0.078
Most of the time	227 (87.3)	91 (82.0)	136 (91.3)	-	
Sometimes	7 (2.7)	5 (4.5)	2 (1.3)	-	
Never	26 (10.0)	15 (13.5)	11 (7.4)	-	
**Access to alcohol/gel at work for hand hygiene *N* (%)**					<0.001
Most of the time	211 (81.1)	73 (65.8)	138 (92.6)	-	
Sometimes	9 (3.5)	6 (5.4)	3 (2.0)	-	
Never	40 (15.4)	32 (28.8)	8 (5.4)	-	
**Partner’s work sector *N* (%)**					<0.001
Floriculture	119 (40.6)	51 (58.6)	23 (22.5)	45 (43.3)	
Agricultural work	19 (6.5)	14 (16.1)	2 (2.0)	3 (2.9)	
Other	155 (52.9)	22 (25.3)	77 (75.5)	56 (53.8)	
**D. Urinary ETU_SG_ metabolite levels (µg/L)**					
					0.036
N	406	109	149	148	
Geometric mean	3.38	5.61	3.07	2.57	
Mean	6.84	10.07	5.94	5.37	
Minimum	0.28	0.28	0.40	0.34	
25th quantile	1.59	3.25	1.47	1.38	
Median	3.25	5.27	2.89	2.33	
75th quantile	5.95	10.56	5.10	3.92	
90th quantile	13.60	18.79	12.53	9.84	
Maximum	196.84	122.40	116.38	196.84	

^1^ Sample size for characteristics = total (*N* = 409); agricultural workers (*N* = 111); non-agricultural workers (*N* = 149); non-workers (*N* = 149), unless stated otherwise. ^2^ Majority floriculture workers (*N* = 102).

The mean maternal age of the sample was 27 (SD = 5.8) years, with the mean gestational age at baseline being approximately 15 (SD = 3.2) weeks. Most participants resided in the Cayambe canton at the time of the baseline interview (75%), and, on average, participants reported living in the region for over a decade. To assess socioeconomic status, we created a scale for overall ownership of material goods. The material ownership scale includes YES/NO (scored as 1/0) answers for ownership of television, computer, internet (WiFi and cell phone), residential telephone, cellular telephone, smartphone, refrigerator, car, gas stove, electric stove, washing machine, and shower with hot water (range: 0, 13). On average, participants reported approximately 7 items on the material ownership scale, with non-workers reporting significantly less compared to the two worker groups (*p*-value: <0.001). Over three quarters of the sample reported either being married or in a domestic partnership (77%) and reported living with their partner/husband (77%), with a larger percentage reported among agricultural workers for both variables. A majority reported living with children, with the largest proportion among agricultural workers (*p*-value: 0.001). Maternal education levels were classified according to the categories established by the Ecuadorian government and then converted into total years of education [35]. On average, participants reported a total of approximately 12 years of schooling, with levels varying significantly by work sector (*p*-value < 0.001). The partner’s education level followed a similar pattern. Households had, on average, two income earners, although non-workers reported fewer earners (*p* < 0.001). The mean monthly household income was $512 (SD = 227), supporting approximately four household members. Non-workers reported a lower income ($404; *p* < 0.001), while the number of dependents remained similar across all groups. Across all groups, husbands or partners were the main income providers. Participants were asked about financial difficulties in covering basic needs, rent, utilities, and education expenses. A composite scale was developed to measure overall financial strain and hardship. This scale includes YES/NO (scored as 1/0) answers for difficulty meeting the cost of food/other basic needs, housing payments, utilities, phone bills, clothing, education, health services, and other needs (range: 0, 8). Non-workers reported a significantly higher financial hardship scale score than both agricultural and non-agricultural workers (*p*-value: 0.0054). Most participants identified as Mestiza (77%); however, a higher proportion of more agricultural workers identified as Indigenous compared with the other two groups.

Residential environmental characteristics are presented in Table 1B. Most participants reported having a toilet with a sewage system (80%), and access to piped potable water for domestic use (96%) and for consumption (96%). Just under a quarter of the sample reported living near an irrigation ditch or open water canal, with an average distance of approximately 17 m. Approximately one third of participants reported having a plot for domestic cultivation, with similar distributions across work sector group. Just over a third of the participants reported being responsible for those domestic plots (36% overall). About 14% of the participants reported using chemicals on their domestic plots, with agricultural workers reported significantly more usage than non-agricultural or non-worker participants (27%). Because of the high use of fungicides, particularly mancozeb, on certain foods including potatoes and tomatoes (staple foods in the region) and the potential for degradation of EBDCs into ETU as part of the cooking process [36], participants were asked about frequency of consumption of these foods in the week prior to the interview and urine collection. Participants reported consuming potatoes an average of 7.1 times per week (SD = 5.8), with a non-significant trend toward higher consumption among agricultural workers (*p* = 0.075). Kidney tomato consumption averaged 4.8 times per week (SD = 4.1), with no significant differences between groups (*p* = 0.483). Tree tomato consumption was the least frequent (M = 1.7, SD = 2.8) and showed no significant variation across groups (*p* = 0.702). Finally, participants were asked about air quality outside of their home and about half reported that the air quality was not clean and odorless, with similar proportions across groups.

Specific occupational characteristics were assessed (Table 1C). Participants reported an average of 38.5 h of work in the week before the interview (SD = 18.8), with no significant difference between groups (*p* = 0.491). Contact with chemical products at work was significantly more common among agricultural workers (27.0%) compared to non-agricultural workers (8.0%) (*p* < 0.001). Most worker participants reported consistent access to water for hand hygiene (93.0%), with no significant group differences (*p* = 0.282). Access to soap was also generally high (87.3%), though agricultural workers were less likely to report having access most of the time (82.0% vs. 91.3%; *p* = 0.078). In contrast, access to alcohol-based hand sanitizers showed a marked difference, with 92.6% of non-agricultural workers reporting having frequent access compared to 65.8% among agricultural workers (*p* < 0.001). Partner occupation varied significantly by group (*p* < 0.001). Participants whose partners worked in floriculture made up 58.6% of the agricultural worker group but only 22.5% of non-agricultural workers. Conversely, partners of non-agricultural workers were more likely to work in other sectors (75.5% vs. 25.3%).

**Figure 1:**
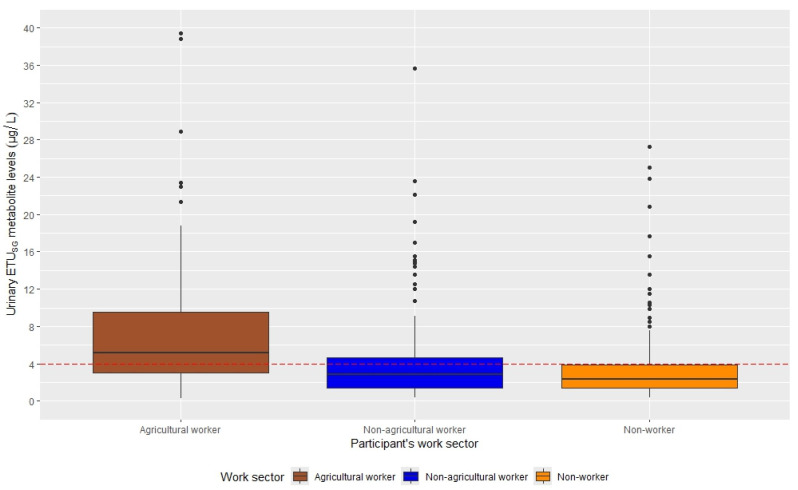
Boxplots of urinary ETU_SG_ metabolite levels across maternal work sector at baseline, SEMILLA Study, Cayambe, Ecuador. 2018–2024.

Table 1D presents descriptive data on urinary ETU_SG_ levels. At baseline, urinary ETU_SG_ levels in the entire sample were elevated (geometric mean: 3.38 µg/L; IQR: 4.36), with 39.4% greater than 4 μg/L. Agricultural workers have significantly higher ETU_SG_ levels compared to the non-agricultural and non-worker groups (geometric mean: 5.61 versus 3.07 and 2.57, respectively; *p*-values < 0.001). Furthermore, the majority of agricultural workers had levels above the >4 μg/L threshold (67%), as shown in Figure 1.

### 3.2. Overall Work Sector Models

We first tested whether the type of work sector affected ETU_SG_ levels (hypothesis 1), adjusting for potential confounders. For our overall population (including both working and non-working participants), the participant’s work sector had a significant effect on log-ETU_SG_ levels, adjusting for relevant covariates (Table 2). Agricultural workers had significantly higher levels of log-ETU_SG_ when compared to non-workers (b = 0.78; M = 1.73 vs. M = 0.95, *p*-value < 0.0001) and when compared to non-agricultural workers (b = 0.6; M = 1.73 vs. M = 1.13, *p*-value < 0.0001); there was no significant difference in log-ETU_SG_ levels between non-agricultural workers and non-workers (b = 0.18; M = 1.13 vs. M = 0.95, *p*-value = 0.1252).

Similarly, the participant’s work sector was related to having ETU_SG_ levels higher than 4 μg/L, adjusting for relevant covariates (Table 2). Agricultural workers had higher odds of ETU_SG_ levels > 4 μg/L when compared to non-workers (OR = 5.43, 95% CI = (3.14, 9.38), *p*-value < 0.0001); non-agricultural workers had higher odds of ETU_SG_ levels > 4 μg/L when compared to non-workers (OR = 1.89, 95% CI = (1.14, 3.14), *p*-value = 0.014); and finally, agricultural workers had higher odds of ETU_SG_ levels > 4 μg/L when compared to non-agricultural workers (OR = 2.87, 95% CI = (1.72, 4.81), *p*-value < 0.0001).

### 3.3. Workers Only Models

**Figure 2:**
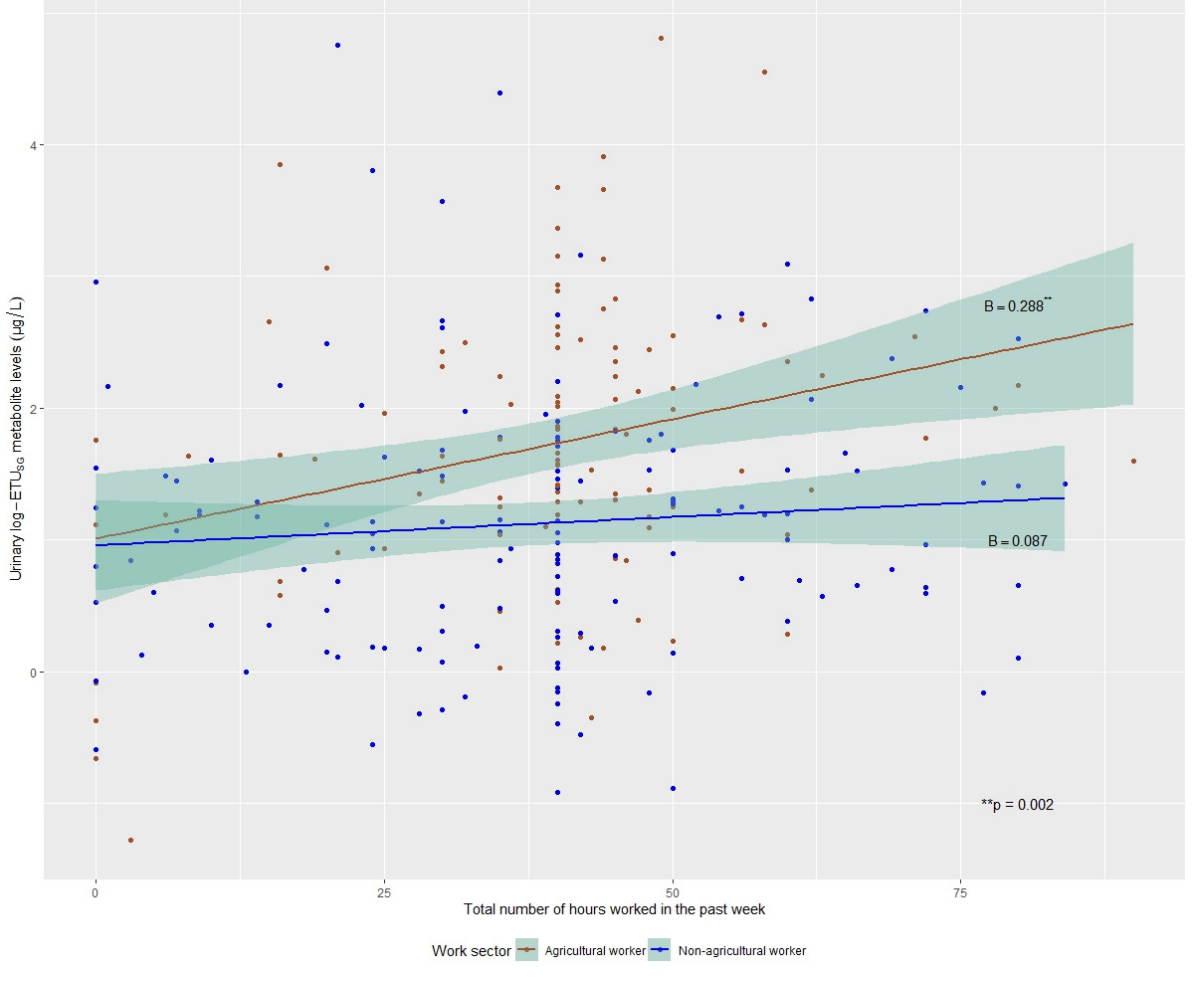
Linear regression models of total hours worked in the past week and natural-log ETU_SG_ levels by work sector, SEMILLA Study, Cayambe, Ecuador. 2018–2024.

Secondly, we sought to test whether work hours were related to ETU_SG_ levels among working participants (hypothesis 2). The participant’s work sector at baseline had a significant effect on log-ETU_SG_ concentration levels. Within the workers-only population (agricultural and non-agricultural workers), we found an association between the number of hours worked in the past week and log-ETU_SG_ levels, varying by the participant’s work sector (*p*-value for interaction = 0.052). For agricultural workers, ETU_SG_ levels were higher for greater work hours in the past week (B = 0.288, 95% CI = (0.105, 0.472), *p*-value = 0.002). For non-agricultural workers, there was no dose–response relationship between hours worked in the past week and ETU_SG_ levels (B = 0.087, 95% CI = (−0.064, 0.249), *p*-value = 0.291). All data points and their subsequent lines of best fit (grouped by their work sector) and CIs, are illustrated in Figure 2.

When assessed as a binomial measure (≤4 μg/L; >4 μg/L), there was no interaction effect between work sector and number of hours worked in the past week in predicting high ETU_SG_ (>4 μg/L), (*p* value for interaction = 0.277). The participant’s work sector had a significant effect on urinary ETU_SG_ levels higher than 4 μg/L, with higher odds among agricultural workers compared to non-agricultural workers (OR = 2.75, 95% CI = 1.64, 4.61, *p* value < 0.001). Similarly, longer work hours in the past week were significantly related to having ETU_SG_ levels higher than 4 μg/L. For every 5 additional hours worked in the past week, the odds of ETU_SG_ levels higher than 4 μg/L increased by 10% (OR = 1.1, 95% CI = (1.03, 1.18), *p* value = 0.008).

## 4. Discussion

The results of this study show elevated urinary ETU_SG_ metabolite levels among all participants of the SEMILLA study, with agricultural workers showing the highest levels of urinary ETU_SG_. Findings show that agricultural work (here, primarily floricultural work) is a strong predictor for elevated ETU_SG_ levels at baseline, even after controlling for other environmental and occupational predictors of ETU_SG_. Results also indicate that a dose–response relationship exists for weekly work hours and ETU_SG_ levels, with more work hours in the past week being associated with higher ETU_SG_ metabolite levels, particularly for agricultural workers.

To our knowledge this is one of the few studies worldwide that has quantified urinary ETU exposure during pregnancy, with a focus on work during pregnancy as an important predictor. Our findings are consistent with the few studies that have investigated the potential for ETU exposure among pregnant women, particularly in agricultural settings and LMIC settings, including prior studies in the region [24]. The urinary ETU_SG_ metabolite levels reported here were much higher than what has been reported for the CHAMACOS pregnancy cohort population [37], a somewhat comparable agricultural community in the U.S. Our findings are also comparable to findings from studies with pregnant nursery workers in Florida [20]. Studies in LMICs are scant. Our findings are comparable to published findings in a pregnant agricultural population in Costa Rica in an industrial agricultural (banana production) setting [21]. Importantly, with the exception of Runkle and colleagues, these studies did not specifically focus on pregnant workers. This is consistent with findings from a recent systematic review of human biomonitoring studies of ETU. In their review, Stadler and colleagues [4] found that very few studies have investigated ETU exposures in environmentally exposed adults, with very few focusing on pregnant women.

Because of the lack of studies in this area, it is challenging to compare our findings with others and draw conclusions regarding differences in quantification of ETU metabolite levels in pregnancy. Our findings support the notion that occupationally exposed pregnant agricultural workers may be at higher risk of ETU exposure with potential health-harming effects to the pregnant worker and the developing fetus and baby.

### 4.1. Limitations

This study has several limitations to note. First, we did not randomly sample participants, but enrolled all eligible women that we could identify, resulting in a convenience sample. Thus, our sample may not represent the ETU exposure distribution for all pregnant women in the region. Second, urine samples were collected at varying gestational ages (8 to 20 weeks), due to broad inclusion criteria, which could have introduced variability in urinary ETU levels related to physiological changes across pregnancy. However, we addressed this limitation by collecting urine samples as first morning void, correcting the ETU metabolite levels for specific gravity, and adjusting for gestational age in the regression models. Third, ETU is rapidly metabolized and excreted from the body, which can make it challenging to capture accurate exposure levels through spot urine samples. However, we addressed this limitation by coordinating the timing of data collection: potential predictors of ETU exposure were assessed via survey within the week preceding urine sample collection. This temporal alignment may help to reduce potential misclassification between exposures reported via survey and measured ETU metabolite levels in urine. Nevertheless, we acknowledge the inherent challenges of using short-lived biomarkers to assess long-term exposure effects. Future work will further explore temporal patterns of ETU levels across pregnancy and investigate exposure pathways specific to floricultural workers, with the goal of better characterizing potential chronic exposure risks. Finally, confounders were selected based on theoretical relevance and prior evidence, and manual stepwise selection was used to refine the model. Although stepwise methods can increase the risk of overfitting, we aimed to reduce this by using a conceptually informed approach. The final model included only predictors that contributed meaningfully, to maintain parsimony and interpretability.

### 4.2. Strengths

Despite these limitations, this study has several important strengths. To our knowledge, this is the first study to specifically examine ETU exposure among pregnant agricultural workers in Ecuador, and one of very few globally, thus contributing important evidence to this field of study. Urine samples were collected from nearly all participants enrolled in the SEMILLA study at baseline, ensuring robust coverage of the cohort and minimizing potential bias. Further, the use of standardized collection procedures including ensuring that all urine samples were collected as FMV helped further reduce potential measurement bias. Moreover, by focusing the analysis on baseline data, we minimized the variability that can occur due to physiological changes across pregnancy. Future analyses will explore within-person variation in ETU metabolite levels across pregnancy to further characterize exposure patterns over time. Finally, our sample population provided a good representation across work sector groups including a large proportion of floriculture workers, which is important for assessing varying levels of occupational and environmental fungicide exposure risk. Future analyses of the SEMILLA data, and additional studies with larger cohorts, could build on these findings to investigate more complex exposure profiles, including co-exposures to other pesticides and contaminants, among vulnerable high-risk groups such as pregnant agricultural workers. While our results demonstrate widespread EBDC fungicide exposure, it is important to consider that agricultural workers are likely exposed to multiple chemicals and other stressors. A more comprehensive assessment of cumulative exposures would offer deeper insight into the environmental health risks faced by vulnerable populations and those who may be at highest risk.

## 5. Conclusions

Global demand for increased food production, alongside the expansion of export-driven floriculture and horticulture industries, has increased the demand for fungicides. Despite their widespread application, substantial gaps remain in our understanding of the developmental and neurobehavioral toxicity of fungicides, particularly EBDCs. Importantly, women of childbearing age may be disproportionately affected, not only because they reside in agricultural communities impacted by large-scale agricultural production, but also because they form an integral part of the agricultural industry labor force in the US and worldwide, particularly in LMICs.

Research quantifying fungicide exposure during pregnancy and examining its effects on infant health and neurodevelopment remains limited, especially for pregnant workers. The findings presented here provide an important initial contribution toward addressing this critical and underexplored area of exposure. Furthermore, although EBDCs are widely used and exposures are increasing, posing a potential risk to maternal and child health, few human studies to date have specifically investigated the effects of prenatal exposure to these fungicides or their primary metabolite, ETU, on newborn thyroid function, early growth, or neurobehavioral development. Future analyses of SEMILLA study data will directly address this important gap in the literature.

## Figures and Tables

**Table 2:** Final model for urinary ETU_SG_ metabolite levels for overall sample, adjusted for key covariates, SEMILLA Study, Cayambe, Ecuador. 2018–2024.

		Estimate for Urinary log-ETU_SG_ Metabolite Levels	*p*-Value	Estimate for Urinary ETU_SG_ Metabolite Levels Higher than 4 µg/L	*p*-Value	Odds Ratio (95% CI)
Intercept		0.852	<0.001	−1.320	<0.001	-
Participant’s work sector at baseline	Agricultural worker	0.780	<0.001	1.692	<0.001	5.43 (3.14, 9.38)
	Non-agricultural worker	0.178	0.125	0.64	0.014	1.89 (1.14, 3.14)
	Non-worker	0	Reference	0	Reference	-
Air quality outside of the home in the past week	Clean, without odor	0	Reference	-	-	-
	Other	0.195	0.050	-	-	-
Frequency of tree tomato consumption in past week	-	-	-	0.010	0.019	1.10 (1.02, 1.19)

## Data Availability

Once the SEMILLA data have been analyzed and main findings are published, we will consider sharing data with qualified researchers, as our study population is vulnerable, and care must be taken to protect subjects. We would consider sharing the data under a data-sharing agreement that documents the users’ commitment to: (1) using the data for specifically defined research purposes agreed upon in advance; (2) providing Institutional IRB approval; (3) securing the data using appropriate computer technology; (4) acknowledgment of the source of data in any publication; and (5) destroying or returning the data after analyses are completed. Requests for data access will be reviewed on a case-by-case basis. Researchers interested in obtaining access should contact the corresponding author for further details.

## Abbreviations

The following abbreviations are used throughout this manuscript:

ETU	Ethylenethiourea
ETU_SG_	Specific gravity corrected ethylenethiourea
EBDC	Ethylenebisdithiocarbamates
SEMILLA	Study of Environmental Exposure of Mothers and Infants Impacted by Large-Scale Agriculture
LMIC	Low- and middle-income country
FMV	First morning void
